# Role of Urotensin-II in Saphenous Vein Graft Disease

**DOI:** 10.21470/1678-9741-2019-0470

**Published:** 2020

**Authors:** Mehmet Erin Tüysüz, Leyla Bahar

**Affiliations:** 1 Department of Cardiovascular Surgery, Mersin City Training and Research Hospital, Mersin, Turkey.; 2 Department of Medical Services and Techniques of Vocational School, Mersin University, Mersin, Turkey.

**Keywords:** Coronary Artery Disease, Diabetes Mellitus, Vena Saphenous, Urotensin-II

## Abstract

**Objective:**

To elucidate the effect of diabetes mellitus (DM) on the atherosclerotic process in saphenous vein grafts by determining urotensin-II (U-II) levels in harvested saphenous veins of patients who underwent coronary artery bypass grafting (CABG).

**Methods:**

Coronary artery disease (CAD) patients who underwent CABG were divided into two groups: Group I (eight non-diabetic patients; CAD group) and Group II (13 patients; DM+CAD group). All patients underwent coronary angiography prior to surgery and Gensini score was used to determine the severity of coronary atherosclerosis. Saphenous vein samples were stained with hematoxylin-eosin and U-II, then damage score, H-Score, and vein layer thicknesses were calculated and statistically evaluated.

**Results:**

In light microscopic evaluation, significant difference was observed between the groups in terms of endothelial cells damage, internal elastic lamina degradation, and tunica media vascular smooth muscle cells (VSMCs) damage (*P*<0.001). U-II immunoreactivity was increased in tunica adventitia in the DM+CAD group (*P*=0.002). The increase in foam cells was directly proportional to the thickening of the subendothelial layer, and this increased U-II immunoreactivity. Gensini score was higher in the DM+CAD group than in the CAD group (*P*=0.002).

**Conclusion:**

Our results show that saphenous vein grafts are already atherosclerotic before they are grafted in CAD patients. This disease is more severe in diabetic CAD patients and these changes can be detected using U-II immunoreactivity.

**Table:** 

Abbreviations, acronyms & symbols				
**AF**	**= Atrial fibrillation**	** **	**IEL**	** = Internal elastic lamina**
**BMI**	**= Body mass index**		**LDL**	**= Low-density lipoprotein**
**BSA**	**= Body surface area**		**NYHA**	**= New York Heart Association**
**CABG**	**= Coronary artery bypass grafting**		**PBS**	**= Phosphate buffered saline**
**CAD**	**= Coronary artery disease**		**SMCs**	**= Smooth muscle cells**
**COPD**	**= Chronic obstructive pulmonary disease**		**SVGD**	**= Saphenous vein graft disease**
**DM**	**= Diabetes mellitus**		**T**	**= Tunica**
**ECs**	**= Endothelial cells**		**U-II**	**= Urotensin-II**
**H&E**	**= Hematoxylin-eosin**		**VLDL**	**= Very low-density lipoprotein**
**HDL**	**= High-density lipoprotein**		**VSMCs**	**= Vascular smooth muscle cells**

## INTRODUCTION

The saphenous vein is the most commonly used graft for myocardial revascularization because of its large diameter, anatomically long course, absence of spasms, and ease of removal^[1,2]^. However, 10% to 20% of saphenous vein grafts fail within one year after coronary artery bypass grafting (CABG)^[3]^. Saphenous vein graft disease (SVGD) significantly affects the short and long-term results of CABG and increases the frequency of major adverse cardiovascular events. It has been shown that many inflammatory and growth factors are secreted from the damaged endothelium in SVGD. These increase the proliferation and migration of smooth muscles in the vascular wall, causing restenosis^[4]^.

Recently, the role of urotensin-II (U-II) in cardiovascular pathophysiology has been researched at large^[5]^. U-II shows its effect by activating the inflammation on the basis of the atherosclerotic process in endothelial cells (ECs)^[6]^. U-II is associated with metabolic regulation and plays an important role in diabetes mellitus (DM) and its complications. Plasma levels of U-II were found to be high in patients with DM who have metabolic syndrome^[7]^.

The aim of our study is to determine the relationship between U-II and atherosclerotic process in the saphenous veins of DM+CAD patients with high Gensini score. As far as we know, this is the first study to report the relationship between saphenous vein atherosclerosis and U-II in the literature.

## METHODS

### Study Design

This prospective study has been approved by the ethics committee of Mersin University (Mersin University Clinical Research Ethics committee 10.09.2018 No: 499). Informed consent was received from the patients included in the study.

The study group included 21 patients who underwent CABG between October 2018 and March 2019 in the Mersin City Training and Research Hospital, Cardiovascular Surgery clinic. Patients were between the ages of 30-80 years. Vein samples (1-2 cm) were obtained from saphenous veins, which were harvested to be used as grafts in CABG.

Inclusion criteria were: having lesion in at least two coronary arteries requiring coronary bypass operation, planned saphenous vein grafting, having no peripheral artery disease (ankle-brachial index: 0.9-1.4), not using venoprotective medications due to venous failure or hemorrhoids, having no recent history of corticosteroid usage, and having no chronic renal failure (uric acid < 4.1-5.0 mg/dL, creatinine <1.2-1.5 mg/dL). Diabetic patients were defined as having fasting blood sugar value ≥ 126 mg/dL and/or HbA1c (%) ≥ 6.4.

### Composing the Experimental Groups

Group 1: Saphenous vein samples obtained from atherosclerotic coronary artery disease (CAD) patients (CAD group: without DM).

Group 2: Saphenous vein samples obtained from atherosclerotic CAD patients (DM+CAD group: with Type 2 DM)

### Surgical Technique

CABG was performed with on-pump technique in all patients. The saphenous veins of all patients were harvested by the same surgeon using the no-touch technique^[8]^. After heparinization, the greater saphenous vein was found immediately proximal to the medial malleolus, the skin incision was made throughout the saphenous vein track, and the vein was completely exposed. Then it was harvested without cannulation or inflating, and then the lateral branches were clipped.

### Histopathological Examination

Prepared saphenous vein grafts (1-2 cm) were taken atraumatically and were immediately put in the fixation solution (10% formalin) in order to perform light microscopic monitoring.

### Tissue Follow-up for Light Microscopic Examination

Tissue samples (saphenous vein parts) were fixed in the 10% formalin solution for 48 hours in the Mersin City Training and Research Hospital’s Pathology Department. The tissues were put into 70% alcohol after their fixation was completed. After the dehydration stage, which was performed by keeping tissues in increasing alcohol ratios (70, 90, 96, 100%), the tissues were treated with three series of xylol for 15 minutes for the transparency step. Before the embedding process, the tissues were kept in soft paraffin for one night. On the next day, saphenous vein samples were blocked in liquid hard paraffin for one hour. Thick sections (5-6 µm) were taken from the paraffin blocks using a microtome (Leica RM-2245). In order to reveal the general characteristics of the saphenous vein tissue, the sections were stained with routine hematoxylin-eosin dye (H&E) and with U-II, in terms of immunoreactivity.

### Immunohistochemistry

The state of the vascular structure in the cardiovascular system was investigated by immunohistochemical methods using the U-II antibody. First, the tissue parts were fixed in 10% formalin, and then embedded in paraffin after routine procedures. A part of the 5-6 µm thick sections was stained with H&E. Routine deparaffinization and rehydration procedures were performed for the sections kept for immunohistochemical staining. After the rehydration stage, they were incubated for 10 minutes with 0.3% Triton X-100 for membrane permeabilization, and then the sections were washed with phosphate buffered saline (PBS pH 7.4) and antigen improvement procedure was performed with citrate buffer (pH 6). To inhibit the endogenic peroxidase activity, 0.03% (vol/vol) hydrogen peroxide solution prepared in PBS was applied to the sections (10 min). Sections were incubated for 18 hours at +4 °C with U-II primary antibody prepared at 1/200 dilution following nonimmune serum administration. Biotinylated secondary antibody and streptavidin-peroxidase enzyme conjugate were administered for 20 minutes, respectively. Three-amino-9-ethylcarbazole chromogen was used in order to occur the color reaction in the sections. To examine the morphology of the tissue, counterstaining was performed by applying Mayer’s hematoxylin for five minutes. Sections which were washed for five minutes under running water were closed with closure solution (Mounting Medium, LabVision, TA-060-UG) and evaluated by light microscope.

### Evaluation of Preparates

After H&E and immunohistochemical staining, the morphological structure of the vascular layers (intima, media, and adventitia), the intact or degenerated cells, and the structure of the internal elastic lamina (IEL) were evaluated in light microscope (Olympus BX53) by considering the morphology of the connective tissue. Examinations were performed using the NIS-Elements Documentation 4.5 image analysis system and photographed with the DP26 camera system. For the calculation of the average number of damaged cells, at least 20 fields of 20x magnification were evaluated in each preparate, and ECs damage, IEL damage, and VSMCs damage in the media layer were scored (none = 0, in focal areas-mild = 1, moderately generalized-severe = 2, generalized-severe = 3, maximum total score = 9). After immunohistochemical staining, the H-score was calculated by evaluating the U-II immunoreactivity positivity in sections. H-score = Ʃ Pi (i+1), where Pi is the percentage of staining in each intensity category (0-100%). The intensity of specific staining was characterized as not present (0), weak but detectable above control (1), distinct (2), very strong (3), and an H-score = 400 is accepted as maximum. In addition, the thickness of intima and media layers were measured and compared.

### Evaluation the Severity of Coronary Stenosis

Patients’ preoperative coronary angiographies were evaluated by two experienced cardiovascular surgeons and a cardiologist before operation.

The severity of CAD was assessed by the modified Gensini score, which was described by Beypınar et al.^[9]^. The score obtained from each degree of stenosis was then multiplied by the coefficient determined according to the lesion’s location in the coronary artery. This scoring was performed for each atherosclerotic lesion in the coronary artery tree, and the results were summed to provide a single value for each patient. The patients with Gensini score < 20% were accepted as mild to absent coronary atherosclerosis and those with Gensini score ≥ 20 % as moderate to severe coronary atherosclerosis^[10]^.

### Statistical Analysis

The statistical analyses were performed using the Statistical Package for the Social Sciences - SPSS software. Numeric variables showing normal distribution were expressed as mean ± standard deviation, and those without a normal distribution were expressed as median (interquartile values). The Mann-Whitney U test was used for two group comparisons. Cases with a *P*-value < 0.05 were considered statistically significant. Power analysis of the research was conducted. Because of the nature of our study (limitations in the human tissue sampling in a prospective study), sufficient number of patients were included in the study according to our power analysis. Assuming type I error, alpha = 0.05, test power = 0.80, the minimum sample width was calculated as 9 for each group of this study.

## RESULTS

### Histopathological Evaluation

The results of light microscopic examination of saphenous vein tissue samples from Group I (control) and Group II (case) were evaluated.

**Fig. 1:**
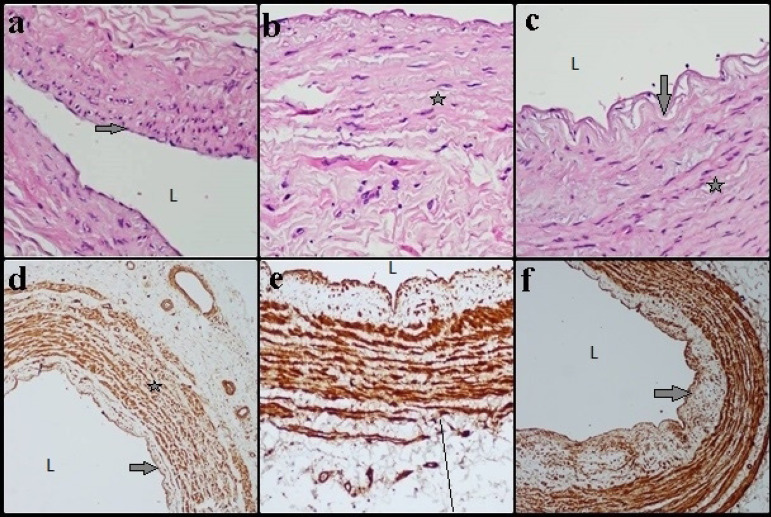
Group I (coronary artery disease): vein sections stained with hematoxylin-eosin (a, b, and c) and parts stained for immunoreactivity with urotensin-II (d, e, and f). Endothelial cells, media layer vascular smooth muscle cells, and internal elastic lamina (IEL) were evaluated as ‘in normal structure’. Down arrow=IEL; right arrow=endothelial cells; star=tunica media. L=lumen. Scale bar=50 µm.

When the sections of the Group I (CAD) were examined, the vein sections stained with H&E - ECs, VSMCs of the media layer, and IEL - were evaluated as ‘in normal structure’ (Figure 1A, B, and C). In U-II stained immunohistochemical sections, it was observed that the tunica intima was regular, however there were fatty streaks and ECs have protected their structure. The subendothelial layer was observed to be thin in most parts. The IEL was observed as indented, while the VSMCs and collagen fibers were exhibiting normal structure. Smooth muscle fibers formed a large number of concentric layers in the media layer, and circular, corrugated, thick fiber bundles were seen adjacent to vasa vasorum. Tunica intima and tunica adventitia layers were stained in moderate intensity in terms of immunoreactivity. Intense-strong immunoreactivity was observed in the tunica media layer (Figure 1D, E, and F).

**Fig. 2:**
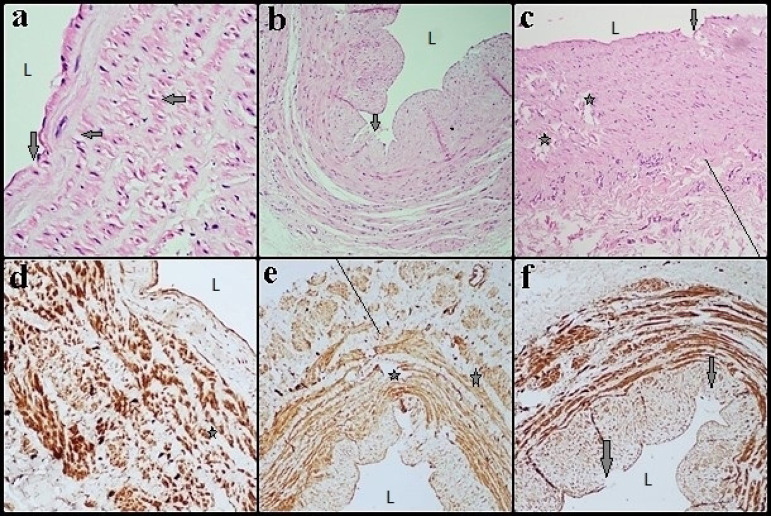
Group II (diabetes mellitus + coronary artery disease): vein sections stained with hematoxylin-eosin (a, b, and c) and parts stained for immunoreactivity with urotensin-II (d, e, and f). (a, b, c, and f) Endothelial areas are visible with lower arrows, internal elastic lamina lost its regular structure, and ruptures occurred in some regions. (d, e, and f) The vascular smooth muscle cells in the tunica media layer could not maintain its normal structure, there were changes in cell morphology and localization in many areas, and there were significant separations in the layer. Left arrows point to foam cells. Star=tunica media. L=lumen. Scale bar=50 µm.

In Group II (DM+CAD), staining of the vascular tissue sections with H&E and U-II revealed that the areas, having lost their continuity, were encountered in the endothelial layer. The ECs were locally damaged and there were destructions in the parts facing the lumen. Examining a large magnification of the ECs, vesicles localized in the apical and basal cell membranes were observed to be more common in the DM+CAD group than in the control group. It was remarkable that the subendothelial layer was locally enlarged and these regions protruded towards the lumen (Figure 2A, B, and C). The VSMCs and collagen fibers were seen to have increased in the subendothelial layer (intimal hyperplasia) and this layer was observed to be thicker than the normal structure compared to the control group (*P*<0.05). Fatty streaks were prominent in the intimal layer. It was observed that IEL lost its regular structure and distructions occurred in some regions. It was noted that VSMCs in the tunica media layer could not maintain its normal structure. Also, there were changes in cell morphology and localization in many areas and there were significant separations in the layer. In terms of immunoreactivity, tunica intima showed moderate staining, but tunica media showed strong staining, and in the tunica adventitia layer, the immunoreactivity positivity was observed to be higher than in the control group (*P*<0.05), (Figure 2D, E, and F).

### Statistical Evaluation

**Fig. 3:**
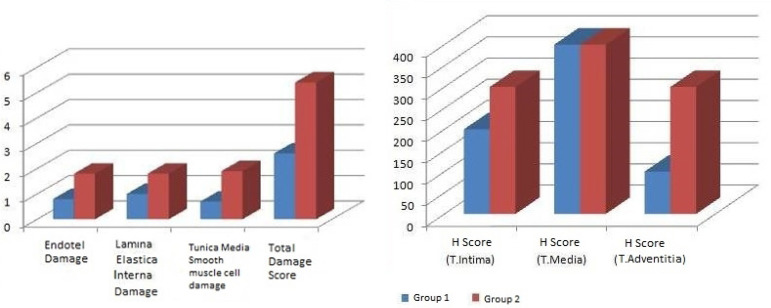
Histological evaluation results of saphenous vein wall structure. As shown in A, there was a significant difference between the groups in the 'total damage score' (P<0.001). In H-score evaluation, there was no statistically significant difference between the groups in terms of tunica (T) intima and T media, however, as shown in B, a significant difference was determined between the groups in terms of T adventitia.

The patients’ body mass index, body surface area, lipid profile, and smoking history were compared using mean ± standard deviations. There were no significant differences between the groups in terms of cardiovascular disease risk factors and individual characteristics (*P*>0.05). However, a significant difference was found between CAD and DM+CAD groups in the Gensini score (*P*=0.006), the details are shown in Table 1. Mann-Whitney U test was used for paired group comparisons. Saphenous vein characteristics between groups were compared according to light microscopic findings. ECs damage, IEL damage, and tunica media SMCs damage were scored; mean, standard deviations, and *P*-values were calculated. Furthermore, there was a significant difference between the groups in total damage score (*P*=0.001) as shown in Figure 3A. In H-score evaluation, there was no statistically significant difference between the groups in terms of tunica intima and tunica media. However, as shown in Figure 3B, a significant difference was found between the groups in terms of tunica adventitia (*P*=0.001) and the details are shown in Table 2. The thickness of intima and media layers of the study groups were measured and compared using Mann-Whitney U test. The vessel tunica intima in the DM+CAD group was thicker than in the CAD group (*P*=0.001), more details are shown in Table 3.

**Table 1:** Comparison between the patients' demographic characteristics and Gensini scores.

Variables	All patients (n: 21)	CAD (n: 8)	DM+CAD (n: 13)	*P*-value
Age (years)	62.7±8.2	59.7±6.8	64.2±8.6	0.25
Gender (male - female)	17 (85.0)	6 (85.7)	11 (84.6)	0.73
	4 (15.0)	2 (14.3)	2 (15.4)	
Height (m)	1.7 (1.7-1.7)	1.7 (1.7-1.7)	1.7 (1.6-1.7)	0.88
Weight (kg)	77 (73-80)	77 (73-84)	76 (72-80)	0.70
BSA (m^2^)	1.9±0.1	1.9±0.1	1.9±0.2	0.75
Waist circumference (cm)	61.4±31.7	75.6±37.2	54.2±27.4	0.19
BMI (kg/m^2^)	27.3±3.3	27.6±1.5	27.1±4.0	0.74
Triglycerides (mg/dL)	166.9±69.6	150.7±57.7	175.6±76.0	0.47
Cholesterol (mg/dL)	165.5±51.9	163.7±52.1	166.7±54.5	0.91
HDL (mg/dL)	39.5±7.1	40.8±3.0	38.9±8.5	0.57
LDL (mg/dL)	92.6±40.9	92.6±45.0	92.5±40.4	0.99
VLDL (mg/dL)	32.9±13.3	30.2±11.6	34.4±14.4	0.51
Creatinine (mg/dL)	0.88±0.41	0.85±0.38	0.98±0.48	0.52
Uric acid	7.4±2.2	6.9±2.1	8.1±2.4	0.25
	**Gensini score category**			
	Gensini score < 20, Gensini score ≥ 20			
**Gensini score**	**All patients (n: 21)**	**CAD (n: 8)**	**DM+CAD (n: 13)**	**0.006**
Mean rank	21.43	6.33	13.27	
Min-max	(12-128)	(12-72)	(33-128)	

BMI=body mass index; BSA=body surface area; CAD=coronary artery disease; DM=diabetes mellitus; HDL=high-density lipoprotein; LDL=low-density lipoprotein; VLDL=very low-density lipoprotein

**Table 2:** Total damage scores and H-score comparisons.

Saphenous vein - urotensin-II	ECs damage (max: 3)	IEL damage (max: 3)	Tunica media SMC damage (max: 3)	Total damage score (max: 9)	H-score = Pi (i+1)		
					Tunica intima	Tunica media	Tunica adventitia
CAD (n: 8)	0.8±0.2	1.0±0.1	0.7±0.1	2.6±0.5	200 (150-250)	400(400-400)	100 (100-200)
DM+CAD (n: 13)	1.8±0.4	1.8±0.2	1.9±0.2	5.4±0.6	300 (200-300)	400(400-400)	300 (300-300)
***P***-value	< 0.001	< 0.001	< 0.001	< 0.001	0.11	0.80	0.002

CAD=coronary artery disease; DM=diabetes mellitus; ECs=endothelial cells; IEL=internal elastic lamina; SMCs=smooth muscle cells

**Table 3:** Tunica intima and media thickness comparison between groups.

Layer thickness of saphenous vein (µm)	T intima thickness (mean±standard deviation)			T media thickness (mean±standard deviation)		
	T intima, minimum	T intima, maximum	T intima, mean	T media, minimum	T media, maximum	T media, mean
CAD	74.75±30.26	131.67±56.03	110.96±40.01	218.35±112.40	264.00±122.17	242.98±117.43
DM+CAD	129.66±34.43	189.65±14.25	155.15±14.23	256.15±82.28	296.72 ±72.85	275.11±59.85
*P*-value	0.001			0.470		

CAD=coronary artery disease; DM=diabetes mellitus; T=tunica

### Clinic Follow-up (One Year)

One year after the operations, all patients were called, and hospital records were viewed. The preoperative and postoperative characteristics of the participants are seen in details in Table 4.

**Table 4:** Preoperative and postoperative baseline characteristics of the study participants.

Preoperative stage results			
Baseline characteristics (n(%))	All patients (n: 21)	CAD (n: 8)	DM+CAD (n: 13)
Hypertension	12 (57.14%)	5 (62.52%)	7 (53.84%)
Hyperlipidemia	9 (42.85%)	4 (50%)	5 (38.46%)
COPD	5 (23.80%)	2 (25%)	3 (23.07%)
Chronic kidney disease	6 (28.57%)	2 (25%)	4 (30.76%)
Family history of CAD	9 (%)	4 (50%)	5 (38.46%)
History of renal failure	2 (15.38%)	0 (0%)	2 (15.38%)
Reoperation (CABG)	0 (0%)	0 (0%)	0 (0%)
Preoperative AF	0 (0%)	0 (0%)	0 (0%)
Smoking status	11 (52.38%)	4 (50%)	7 (53.80%)
**End of the postoperative 1^st^ year stage results**			
Angina pectoris	3 (14.28%)	1 (12.50%)	2 (15.38%)
Re-hospitalization	0 (0%)	0 (0%)	0 (0%)
NYHA class (I-II-III-IV)	1 (%)	0 (%)	1 (%) class I
Mortality	0 (%)	0 (%)	0 (%)

In Table 4, between both groups and in terms of demographic profile there was no significant difference (*P*>0.05). AF=atrial fibrillation; CABG=coronary artery bypass grafting; CAD=coronary artery disease; COPD=chronic obstructive pulmonary disease; DM=diabetes mellitus; NYHA=New York Heart Association

## DISCUSSION

In our study, Gensini score, total damage score, U-II immunoreactivity density, and intima layer thickness of the DM+CAD group were higher than those of the CAD group. Fatty streaks were detected in the saphenous vein walls of both groups.

Cardiovascular functions are regulated through neuronal transmitters, hormones in the circulation, and factors released from local tissues. U-II, a secreted vasoactive peptide hormone of the somatostatin family, is one of them. It provides its effect on the cardiovascular system by stimulating a pair of G proteins^[11]^. The effectiveness of U-II varies according to the vein diameter, vascular bed, and vein type. Its net effectiveness on vascular tonus is to provide a balance between the endothelium-independent vasoconstriction and the endothelium-dependent vasodilatation^[12]^. U-II plays an important role in the development of insulin resistance, in the atherosclerosis, and the glucose metabolism^[13,14]^. Thus, U-II plays an important role in DM and its complications. Plasma levels of U-II were found to be high in DM patients with metabolic syndrome^[15]^. It has been shown that the frequency of glucose intolerance is significantly higher in the CAD group, especially in multivessel diseased patients^[16]^. In our study, we have found that the Gensini score is higher in the DM+CAD group. In this group with higher Gensini score, there was increased U-II immunoreactivity in the harvested saphenous vein wall. This suggests that the high vessel wall damage score in the DM+CAD group is related to the increased effect of U-II on inflammation in diabetic patients.

Another effect of U-II on the atherosclerotic process is to increase the regulation of acyl-coenzyme of macrophages. This increase leads to the conversion of macrophages to foam cell formation. U-II acts on the proliferation of VSMC by activating nicotinamide adenine dinucleotide phosphate and plasminogen activator-1. U-II accelerates the development of atherosclerosis with all these effects^[17]^. We also examined the histopathological effects of U-II on the saphenous vein wall of our patients. In our DM+CAD group with high Gensini score, saphenous veins had high U-II immunoreactivity in the tunica adventitia. This suggests that the inflammatory effect of DM may progress to the outer parts of the vessel, starting from the lumen. In the DM+CAD group, damage of ECs in the intima layer, damage of smooth muscle cells in the media layer, and damage of IEL were found to be higher than in the CAD group. We detected fatty streaks in both groups by light microscope and this showed that saphenous veins were affected by atherosclerosis.

The Coronary Artery Surgery Study revealed that the graft patency was 90% in 60 days and 82% in 18 months^[18]^. However, it was also reported that harvesting the saphenous vein grafts by the conventional technique leads to graft occlusion within the first month in up to 15% of coronary artery bypass patients^[19,20]^. The risk factors that are responsible for SVGD are surgery-related factors, smoking, DM, and hyperglycemia^[21]^. Koshizaka et al.^[22]^ reported the angiographic results of graft failure in one and five years in patients with DM who underwent CABG. According to their study, one-year results showed no difference between diabetic and non-diabetic patients. However, when the five-year results are examined, mortality, myocardial infarction, and revascularization rates are higher in diabetic patients than in the non-diabetics. In our study, in one-year patient follow-up results, there was no difference between the groups in terms of chest pain, re-hospitalization, functional capacity, and mortality. This may be due to the fact that the atherosclerotic process in the saphenous vein has not reached the degree of stenosis that can cause ischemia. Furthermore, atherosclerosis in the saphenous vein is slow and progressive. In addition, we think that saphenous vein preparation with no-touch technique prevents early graft thrombosis by not traumatizing the vein.

### Limitations

Control angiography was not performed in our patients during one-year follow-up due to absence of ischemic symptoms.

## CONCLUSION

We detected that U-II increased in DM patients causes damage to ECs in the intima layer, smooth muscle cells in the media layer, and aggravates inflammation with accumulation of foam cells. Negative effects of U-II in the saphenous vein wall were more significant in the DM+CAD group than in the CAD group.

Multiple studies have shown that SVGD begin shortly after CABG. However, our results show that saphenous vein grafts are already diseased before they are grafted in CAD patients.

**Table:** 

Authors' roles & responsibilities	
MET	Substantial contributions to the conception or design of the work; or the acquisition, analysis, or interpretation of data for the work; drafting the work or revising it critically for important intellectual content; agreement to be accountable for all aspects of the work in ensuring that questions related to the accuracy or integrity of any part of the work are appropriately investigated and resolved; final approval of the version to be published
LB	Substantial contributions to the conception or design of the work; or the acquisition, analysis, or interpretation of data for the work; drafting the work or revising it critically for important intellectual content; agreement to be accountable for all aspects of the work in ensuring that questions related to the accuracy or integrity of any part of the work are appropriately investigated and resolved; final approval of the version to be published
